# Research on Robot Screwing Skill Method Based on Demonstration Learning

**DOI:** 10.3390/s24010021

**Published:** 2023-12-19

**Authors:** Fengming Li, Yunfeng Bai, Man Zhao, Tianyu Fu, Yu Men, Rui Song

**Affiliations:** 1 The School of Information and Engineering, Shandong Jianzhu University, Jinan 250101, China; lifengming21@sdjzu.edu.cn; 2 The School of Control Science and Engineering, Shandong University, Jinan 250061, China; baiyunfeng@mail.sdu.edu.cn (Y.B.); 201934540@mail.sdu.edu.cn (M.Z.); ymen@mail.sdu.edu.cn (Y.M.)

**Keywords:** robot screwing, GMM-GMR, learning from demonstration, dynamic movement primitive

## Abstract

A robot screwing skill learning framework based on teaching–learning is proposed to improve the generalization ability of robots for different scenarios and objects, combined with the experience of a human operation. This framework includes task-based teaching, learning, and summarization. We teach a robot to twist and gather the operation’s trajectories, define the obstacles with potential functions, and counter the twisting of the robot using a skill-learning-based dynamic movement primitive (DMP) and Gaussian mixture model–Gaussian mixture regression (GMM-GMR). The hole-finding and screwing stages of the process are modeled. In order to verify the effectiveness of the robot tightening skill learning model and its adaptability to different tightening scenarios, obstacle avoidance trends and tightening experiments were conducted. Obstacle avoidance and tightening experiments were conducted on the robot tightening platform for bolts, plastic bottle caps, and faucets. The robot successfully avoided obstacles and completed the twisting task, verifying the effectiveness of the robot tightening skill learning model and its adaptability to different tightening scenarios.

## 1. Introduction

Robots operating in industrial production environments often encounter repetitious tasks that are predominantly performed manually, such as screw assembly [1,2]. However, the significant labor costs associated with manual screwing limit the production efficiency for products [3,4]. To address this challenge, teaching robots the skill of screwing becomes crucial, as it offers the potential to enhance production efficiency and quality through the robots’ perception and generalization abilities, as well as their ability to swiftly transition between related activities.

The process of robot screwing involves three primary steps: finding holes, feeding, aligning, and screwing [5,6]. Extensive research has been conducted to explore the tightening process of robots, focusing on state-detection-based models, process-based models, and combinations of process detection and control [7,8]. However, several challenges persist in robot tightening tasks. These challenges include the limited applicability of the existing models, the need for the further development of tightening experience, and the large time costs associated with manual alignment and repositioning during activity transfer.

Learning from demonstration (LFD) refers to a robot learning to analyze the mapping between actions in order to imitate human behavior in a specific task [9]. Learnability and stability are two features of robot skill learning [10]. New robots can quickly learn to complete tasks by learning from expert experience [11]. Robots’ generalization capacity to learn comparable tasks could be considerably increased.

LFD is a method in which a robot is trained to observe the mapping between actions while performing a taught task. Behavior acquisition, behavior representation, and behavior reproduction are three phases with a similar meaning [12,13]. Dynamic movement primitives (DMP), Gaussian mixture models (GMM), and other learning generalization approaches are commonly employed [14]. The fundamental difference between these models lies in their handling of the twisting problem. A DMP breaks down operational skill aspects into motion primitives, which are then combined to produce various actions [15]. GMM-based robot learning skills adopt probability calculation methods, which include extracting relevant skill features and learning the mapping relationships among relevant skill feature information [16]. To achieve robot skill learning, the GMM model is paired with the Gaussian mixed regression model (GMR) [17].

The DMP and GMM-GMR methods are more widely used as a result of their promotion [18,19,20]. Muelling K et al. [21] established a library of action primitives through physical interaction with humans in 2012, and they then extended this to teaching robots to play table tennis. Gams A’s team [22] refined the DMP algorithm for human–robot cooperative handling by allowing the robot to modify its position in response to human force. In 2018, Peternel L’s team used a remote control device to connect a human sensorimotor system to a robot control circuit [23]. A DMP builds force and stiffness throughout the tightening process and solves the challenge of sliding bolt fittings into grooves and assembling them into materials with unknown properties. The paper in [24] recorded motion data from demonstrations of expert surgeons, who performed three surgical tasks; GMM encoded the expert’s basic kinematic structure, and then GMR was used to extract smoothed reference trajectories to reproduce the task trajectories through automatic skills. LFD methods such as DMP and GMM-GMR have been applied to multiple types of task learning and generalization [25,26], but little research has been performed on robotic screwing. Lin et al. developed a smart hand exoskeleton for the case of learning to play musical instruments, to help disabled people to learn dexterous operation skills [27].

LFD-based methods enable robots to repeat simple predetermined behaviors away from constrained environments [28,29] and also perform the optimal actions in unstructured environments [30]. Therefore, modeling thread connection skills based on demonstration learning could be beneficial in improving the learnability and universality of robot thread connection operations and for reducing the deployment costs when the operating environment changes.

The main research is outlined as follows:

1. A framework for the learning of robot screwing skills based on the LFD method is constructed. For the hole-finding process and the screwing process of robot screwing, a robot hole-finding strategy based on the DMP method and the screwing skill learning framework based on GMM-GMR are established;
2. A hole-finding strategy based on DMP and an artificial potential field is proposed. The potential field function of obstacles is added to the DMP learning model to establish a robot hole-finding strategy with the smallest error;
3. A method for the learning and generalization of screwing skills based on GMM-GMR is proposed. We collect screwing teaching information, use dynamic time warping (DTW) for data alignment and then use the GMM method to extract screwing features, perform GMR regression fitting, and filter out smooth screwing characteristic curves;
4. A robot screwing platform is built and the skill generalization is verified. The effectiveness of the robot screwing method based on LFD is verified by considering the tendency and screwing of robot bolts, the trend of screwing plastic bottle caps, and the trend of obstacle avoidance and the screwing of faucets. It is verified that the method can be generalized for different objects and scenarios.

## 2. Method

**Figure 1:**
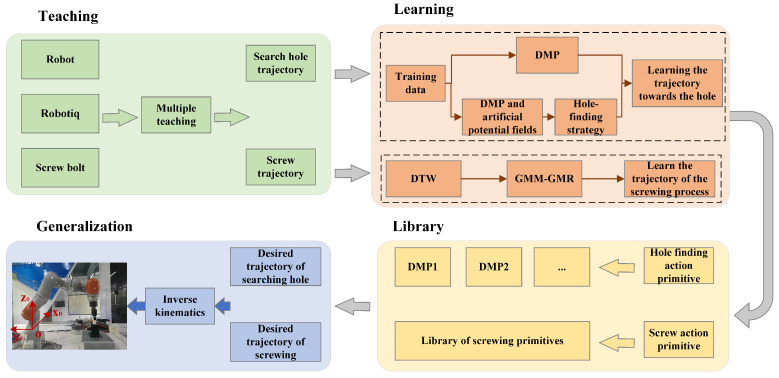
Framework for robot screwing skill training.

The three steps of skill learning include behavior acquisition, behavior representation, and behavior reproduction. Figure 1 illustrates the framework for the screwing skill training of a robot. For screwing teaching, the framework includes modifying the communication between the robot and the gripper. A skill learning model is built for the two fundamental screwing operations, namely hole searching and screwing. The corresponding skill library is obtained through learning, which can then be generalized to other screwing scenes and objects.

LFD is a method that involves training robots to observe the mapping between actions while performing teaching tasks. Behavior acquisition, behavior representation, and behavior reproduction are the three stages.Based on LFD, we conducted research on the relevant methods.

In the process of industrial robot screw assembly, the main steps include approaching the screw assembly target, alignment, and locking. Path planning and obstacle avoidance towards the goal of spiral assembly are the first steps in the spiral operation, which is of great significance for the success of the entire assembly. This study applied the DMP method to skill learning towards the spiral assembly target, dividing the teaching trajectory into discrete motion elements for the trajectory approach and planning. We added a potential field function for obstacles and designed a learning strategy during the hole-finding process. When obstacles appear during trajectory execution, the robot avoids collisions and ensures that the task is executed normally, with minimal error.

After alignment, the task of modeling bolt tightening was carried out. The data of the demonstration dragging robot bolt tightening were preprocessed, and then DTW was used for data alignment. GMM was used for data classification and the characterization of tightening skills, and GMR was used for data regression fitting to obtain a smooth tightening characteristic curve, achieving robot bolt tightening with different starting and ending points and different bolt types.

### 2.1. Screwing Skill Learning

Communication between the robotic arm and the gripper was established. In the teaching process of the robot, we dragged the robot and taught it to complete the screwing task. The screwing skills of different movements were represented by the pose sequence of the robot end. A coordinate system o−xyz was established according to the base of the manipulator. We dragged the teaching robot many times to perform screwing, obtained the hole-finding and screwing trajectory, and used the pose $\left(x_{t},y_{t},z_{t},\alpha_{t},\beta_{t},\gamma_{t}\right)$ of the end of the robot relative to the base coordinate system, where t={1,2,⋯,T} represents the time series of the whole process, and $\left(x_{t},y_{t},z_{t},\alpha_{t},\beta_{t},\gamma_{t}\right)$ is the relative position of the end of the robot.

### 2.2. Hole-Finding Trajectory Learning and Obstacle Avoidance Strategy Based on DMP

We taught and learned to collect the hole-finding trajectory of the robot and passed it to the DMP model. The DMP method was used to convert the linear system into a nonlinear system as follows:

(1) $$\left[\begin{array}{c} \dot{z}\\ \dot{y}\\ \dot{x} \end{array}\right]=\left[\begin{array}{c} (\alpha_{y}\left(\beta_{y}\left(y^{g}-y\right)-z\right)+x\cdot f\left(x\right))/\tau \\ z/\tau \\ -\alpha_{x}x/\tau \end{array}\right],$$

where τ is a time constant. α and β are positive constants representing the stiffness and damping of the spring. The gating restriction term is *x*. The taught trajectory sequence is *y*. The velocity sequence is *z*. The initial state is $y_{0}$, and the target state is $y^{g}$. f(x) is shown as

(2) $$f\left(x\right)=\frac{\Sigma_{i=1}^{N}\psi_{i}\left(x\right)w_{i}}{\Sigma_{i=1}^{N}\psi_{i}\left(x\right)}x\left(y^{g}-y_{0}\right),$$

(3) $$\psi_{i}\left(x\right)=exp\left(-h_{i}{\left(x-c_{i}\right)}^{2}\right).$$

The initial position is and the target position is $y^{g}$. The weight of the basis function is $w_{i}$, and the number of Gaussian basis functions is *N*. A Gaussian function centered on is defined in Equation (3), where $h_{i}$ is the variance. When the *x* of the canonical system converges to its target, the corresponding Gaussian function is activated, causing the forcing function to take effect. The weighted sum of Gaussian functions is normalized and then multiplied by $x(y^{g}\;-\;y_{0})$. In this way, the path towards the screw assembly target can be encoded by a sequence of motion primitives consisting of target points.

The weight corresponding to each function is solved by LWR. The expression can be obtained as follows:

(4) $$w_{i}=\frac{s^{T}\psi_{i}f_{target}}{s^{T}\psi_{i}s},$$

where $s=\left(\begin{array}{c} x_{t_{1}}\left(y^{g}-y_{0}\right)\\ \vdots \\ x_{t_{p}}\left(y^{g}-y_{0}\right) \end{array}\right)$, $\psi_{i}=\left(\begin{array}{ccc} \psi_{i}\left(t_{0}\right) & \ldots & 0\\ 0 & \ddots & 0\\ 0 & \ldots & \psi_{i}\left(t_{p}\right) \end{array}\right)$.

We specified the starting point and target point of the hole-finding trajectory and input it into the DMP model. According to the learned weight parameters, the formula of the dynamic system was calculated, and the position, velocity, and acceleration were obtained through step-by-step integration. The corresponding angle of each joint of the robot was reversely solved by inverse kinematics from the pose in the Cartesian coordinate system, so that the corresponding screwing skills were reproduced.

In order to prevent the end point and the start point from being too close, affecting the calculation of the forcing term, a coupling term $x(y^{g}-y_{0})$ related to the start point and the target point was added on the basis of Equation (1). Then, we added the coupling term φ(y,z) related to the obstacle, and the equation of the changed DMP is as follows:

(5) $$\tau \dot{z}=K\left(y^{g}-y\right)-Dz-K\left(y^{g}-y_{0}\right)x+Kf\left(x\right)+\varphi \left(y,z\right).$$

In general, the coupling term φ(y,z) represents the outward repulsion generated by the obstacle at position *y* to the trajectory. This depends on the position *y* and velocity *z* of the system.

**Figure 2:**
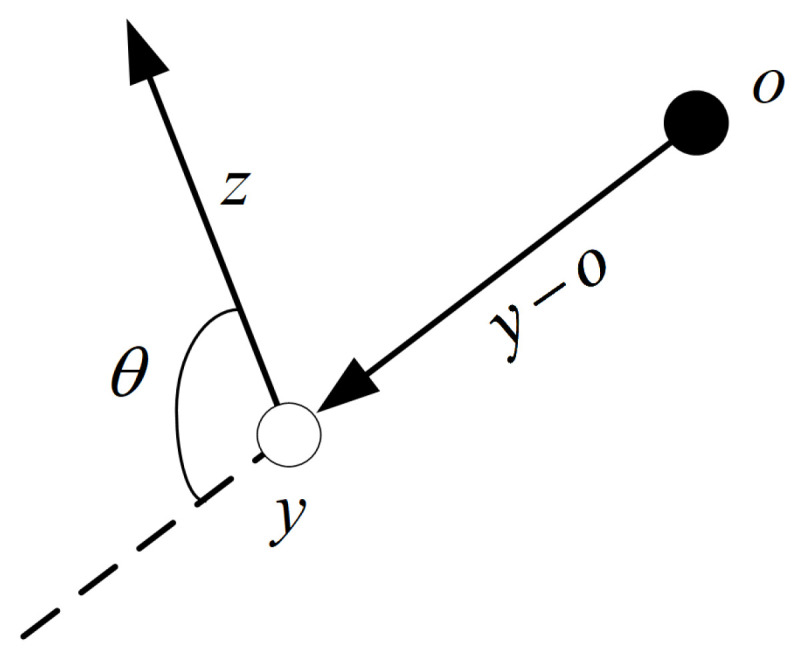
The angle between the current speed and position of the system relative to the obstacle.

There are two main methods of obstacle avoidance based on the artificial potential field: point obstacle avoidance and volume obstacle avoidance, which include static and dynamic obstacle avoidance, respectively. Point obstacle avoidance is used to extract the features of obstacles into points and combine the extracted point information to establish a corresponding potential field, which is coupled to the corresponding dynamic system equation. When the state of the system changes to the range of the obstacle, the coupling function will push the system away from the range of the obstacle and adjust its trajectory. The dynamic potential function is

(6) $$U_{d}\left(y,z\right)=\left\{\begin{array}{l} \begin{array}{cc} \lambda {\left(-\cos\theta \right)}^{\beta }\frac{∥z∥}{p(y)} & \theta \in \left(\frac{\pi }{2},\pi \right] \end{array}\\ \begin{array}{ccccccccc} 0 & & & & & & & & \theta \in \left[0,\frac{\pi }{2}\right]. \end{array} \end{array}\right.$$

(7) $$\cos\theta =\frac{\langle z,y-o\rangle }{∥z∥p\left(y\right)}.$$

where $\lambda ,\beta \in R_{+}$ is a constant gain; $p\left(y\right)\in R_{+}$ is the distance between the obstacle and the system position. θ is the angle between the current speed *z* and position *y* of the system relative to the obstacle, which is shown in Figure 2. Similarly, volume obstacle avoidance is used to extract the information of obstacles in the form of geometry and add them to the coupling term.

**Figure 3:**
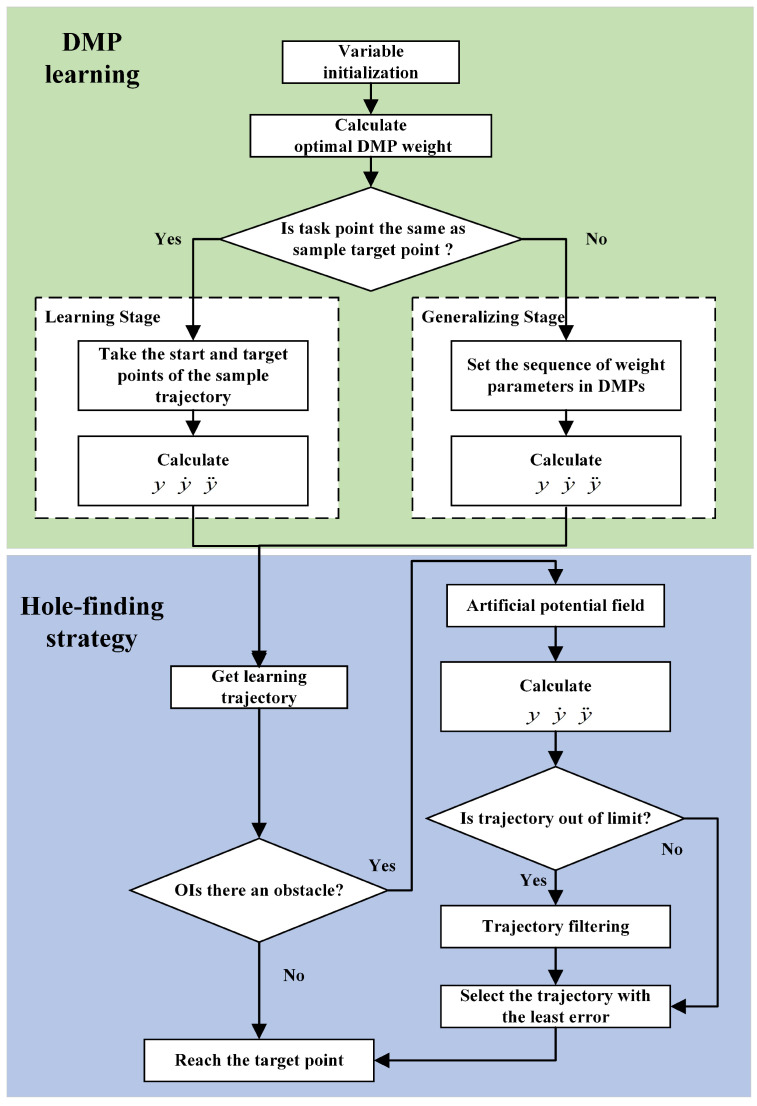
Flow chart of the whole screw-finding experiment.

Combining the motion range of the robot and the obstacle avoidance error formed by different potential functions, a DMP-based obstacle avoidance strategy that allows the robot to find holes in complex environments was proposed. A flow chart of the whole screw-finding experiment is shown in Figure 3.

The system initializes and learns the optimal weight parameters for DMP. Learning and generalization can be performed by judging whether this task is the same as the teaching task. When there is an obstacle, adding an artificial potential field to the trajectory learned by DMP can generate an outward thrust in the range where the obstacle is located and push the trajectory away from the range of the obstacle, thereby achieving the effect of obstacle avoidance. After solving the obstacle avoidance trajectories under various artificial potential fields, we determined whether there was a situation wherein the robot exceeded the limit. We screened out the trajectories that did not exceed the limit and selected the one with the smallest error for reproduction.

### 2.3. Screwing Trajectory Learning with GMM-GMR

The mean value series of the demonstration trajectories were selected as reference series. Dynamic time warping (DTW) was used to determine the similarity between each pose and its mean sequence and align the corresponding similar points between trajectories. Then, GMM-GMR performs feature extraction and fitting regression.

To align two sequences, it is necessary to construct a matrix grid. The matrix elements (i,j) represent the distance $d(q_{i},c_{j})$ between $q_{i}$ and $c_{j}$. We used the Euclidean distance $d\left(q_{i},c_{j}\right)=\sqrt{{\left(q_{i}-c_{j}\right)}^{2}}$ to represent the similarity between the points in the sequence. The smaller the distance, the higher the similarity. Using the dynamic programming (DP) method to find a path through several grid points in this grid, the grid points that the path passes through are aligned points calculated using two sequences.

We set the *j*-th demonstration data point to $\xi_{j}=\left\{\xi_{s,j},\xi_{t,j}\right\}$, j={1,2,⋯,P}, where *P* is the number of data points included in a single teaching, $\xi_{s,j}$ is the spatial coordinate value or node angle, and $\xi_{t,j}$ is the time value. Assume that each data point follows the following probability distribution:

(8) $$p\left(\xi_{t,j}\right)=\sum_{k=1}^{K}p(k)p\left(\xi_{j}|k\right).$$

In Equation (8), p(k) is the prior probability, and $p(\xi_{j}|k)$ is the conditional probability distribution, which obeys a Gaussian distribution. *K* is the number of Gaussian distributions that make up the Gaussian mixture model and *D* is the dimension of the GMM encoding the teaching data.

(9) $$p\left(k\right)=\pi_{k},$$

(10) $$p\left(\xi_{j}|k\right)=N\left(\xi_{j};\mu_{k},\sum_{k}\right)=\frac{1}{\sqrt{{\left(2\pi \right)}^{D}\left|\sum_{k}\right|}}e^{-\frac{1}{2}\left({\left(\xi_{j}-\mu_{k}\right)}^{T}{\sum_{k}}^{-1}\left(\xi_{j}-\mu_{k}\right)\right)}.$$

Therefore, the parameters $\left\{K,\pi_{k},\mu_{k},\sum_{k}\right\}$ are the number of components of the GMM and the prior probability, expectation, and variance of the *k*-th component. The *K* parameters were estimated using the Bayesian information criterion, and a model was selected to achieve a trade-off between model complexity and the optimal data fitting performance. Then, we used the EM algorithm to estimate the parameters $\left\{\pi_{k},\mu_{k},\sum_{k}\right\}$ and finally realize the construction of the model.

The processed data of GMM were reconstructed with GMR. It is known that $p(\xi_{j}|k)$ satisfies a Gaussian distribution $\left(\begin{array}{c} \xi_{s,k}\\ \xi_{t,k} \end{array}\right)∼N\left(\mu_{k},\sum_{k}\right)$, where $\mu_{k}=\left\{\mu_{s,k},\mu_{t,k}\right\}$, $\sum_{k}=\left\{\begin{array}{cc} \sum_{s,k} & \sum_{st,k}\\ \sum_{ts,k} & \sum_{t,k} \end{array}\right\}$. $\xi_{t,k}$ is given and also satisfies the Gaussian distribution, $\xi_{s,k}|\xi_{t,k}∼N\left(\mu_{s,k}^{\prime },\Sigma_{s,k}^{\prime }\right)$, $\mu_{s,k}^{\prime }=\mu_{s,k}+\Sigma_{st,k}{\left(\Sigma_{t,k}\right)}^{-1}\left(\xi_{t,k}-\mu_{s,k}\right)$,$\Sigma_{t,k}^{\prime }=\Sigma_{s,k}+\Sigma_{st,k}{\left(\Sigma_{t,k}\right)}^{-1}\Sigma_{st,k}$.

The mean value $\mu_{s}^{\prime }$ and variance $\Sigma_{s}^{\prime }$ with *K* Gaussian components can be obtained.

(11) $$\eta_{k}=\frac{p(\xi_{i}|k)}{\sum_{i=1}^{K}p(\xi_{i}|i)},\mu_{s}^{\prime }=\sum_{k=1}^{K}\eta_{k}{\mu^{\prime }}_{s,k},\Sigma_{s}^{\prime }=\sum_{k=1}^{K}\eta_{k}^{2}\Sigma_{s,k}^{\prime }.$$

Suppose that the regression function of the GMM is $m(\xi_{t})$

(12) $$m\left(\xi_{t}\right)=E\left(\xi_{s}|\xi_{t}\right)=\sum_{k=1}^{K}\eta_{k}\mu_{s,k}^{\prime }=\mu_{s}^{\prime }$$

## 3. System and Platform

**Figure 4:**
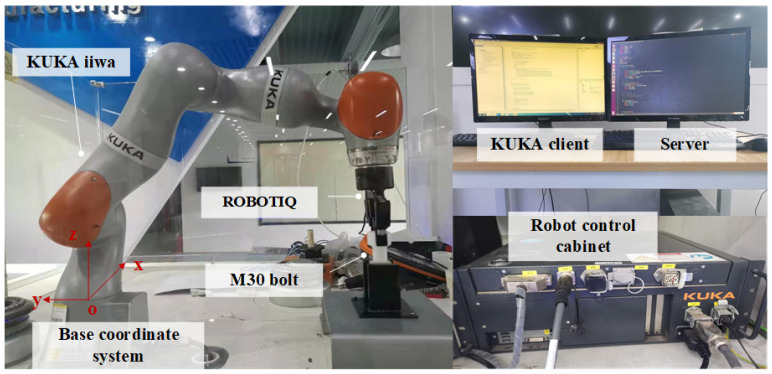
KUKA iiwa robot screwing experiment platform.

To verify the learning model for robot screwing skills, a physical verification platform for robot screwing was built. The base frame of the robot was used as the reference coordinate system for modeling. The platform is shown in Figure 4.

The KUKA iiwa 7 R800 robot in the hardware part has seven degrees of freedom and requires a computer with KUKA Sunrise. The OS controls the robot. The server collects and processes information. The ROBOTIQ 2-FINGER 85 gripper serves as an end-effector that can hold different tightened objects. Different types of bolts, plastic bottle caps, and faucets were considered as screwed objects, which were fixed on the screw base.

A bolt tightening system was built. The control device and the server were used for the position and posture information processing and action decision making of the robot. The robot adjusts to the corresponding screwing posture. The end gripper grasps and tightens the bolts. First of all, the robot, controlled by the KUKA Sunrise.OS 1.16 software, could receive drag teaching and adjust compliance appropriately, so that it could be dragged smoothly and effortlessly. At the same time, the communication between the gripper and the robot was established in the controller.

When reaching the initial position, the gripper was closed and the top of the bolt was clamped. The gripper was opened when the screwing ends. The robot communicated with the server through the Socket protocol, and the pose sequence of the end of the robot in the screwing phase was recorded. After the skill had been learned, the control device sent action instructions to the robot through Socket communication. The robot completed the reproduction and generalization of the screwing skills.

## 4. Experiment and Results

### 4.1. Bolt Finding and Screwing

**Figure 5:**
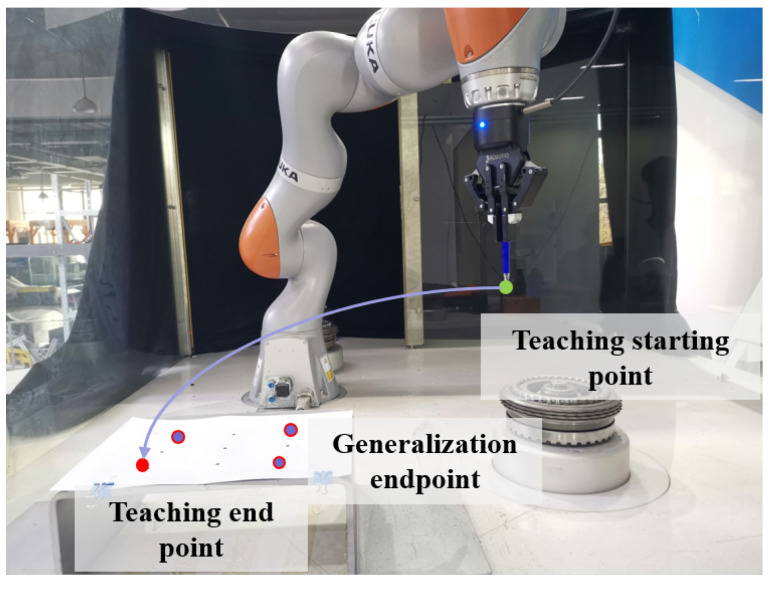
Experimental diagram of teaching hole finding.

We designed a robot hole-finding experiment as shown in Figure 5. We obtained the robot teaching trajectory, conducted hole-finding teaching, and used preprocessed data to filter out singular points. Then, we learned the DMP model and saved the learned weight parameters. We kept the starting point unchanged and set different end points.

The error of DMP was calculated by dividing the distance between the end point and the target point by the Euclidean distance between the end point and the starting point, as shown in (11).

(13) $$error=\frac{dis(\mathrm{Target}-\mathrm{Actual}\mathrm{end}\mathrm{point})}{dis(\mathrm{Target}-\mathrm{Starting}\mathrm{point})}\times 100\%$$

**Figure 6:**
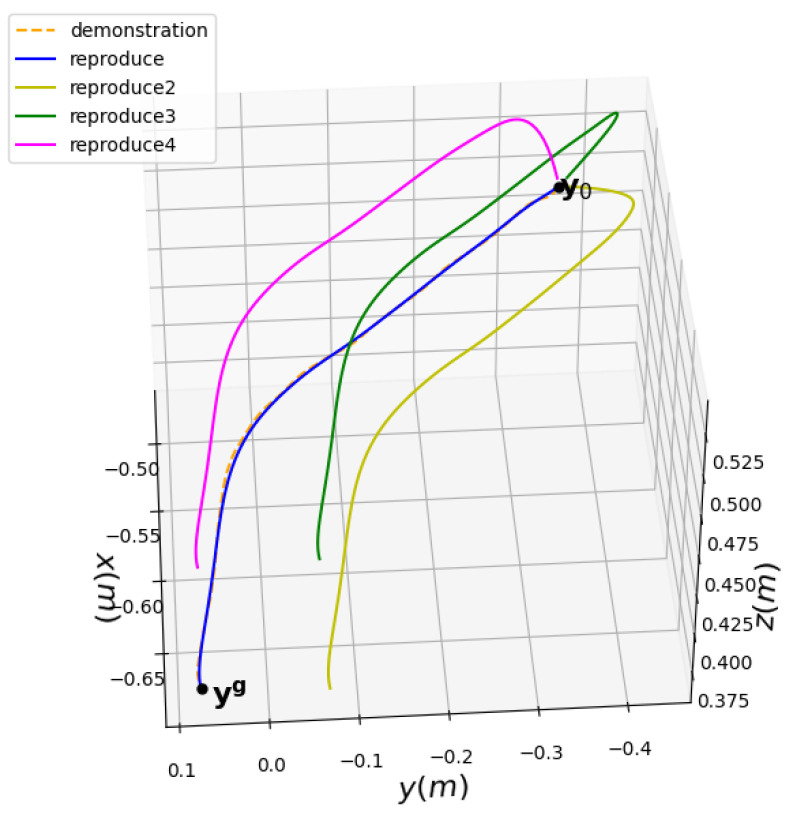
Experimental result of learning and generalization of positions X, Y, Z using DMP.

The error of the generalization trajectory of the transformed target point is shown in Table 1 and an experimental diagram of the generalization trajectory of the transformed target point is shown in Figure 6.

**Figure 7:**
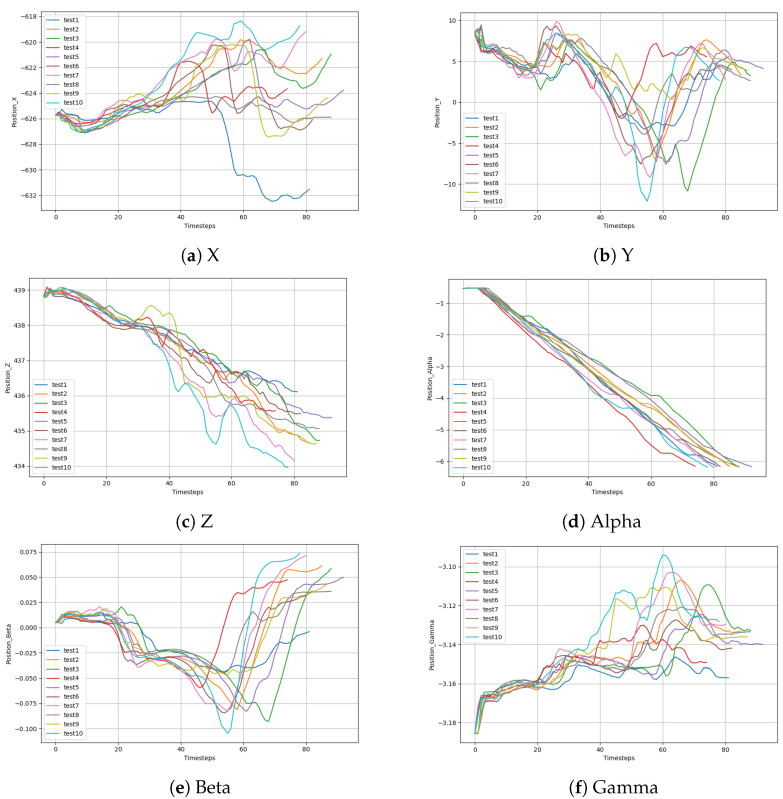
Data for positions X, Y, and Z, and postures Alpha, Beta, and Gamma diagram during the robot screwing process.

The selected bolts were M30 × 3.5 nylon bolts. The outer diameter of the thread was 30 mm and the longitudinal distance between the two threads was 3.5 mm. The drag teaching was performed 10 times, and the pose of the end of the robotic arm was collected every 0.1 s. Due to the variation in drag speeds each time, the length of the data for 300° screwing with the robot varied from 75 to 93. The data were preprocessed and the mutation points and singular points were removed. The length of the data was adjusted to 75 using the equidistant deletion method. As shown in Figure 7, the six figures correspond to the end positions, respectively. The thick blue solid line in the figure represents the mean value of ten taught traces.

**Figure 8:**
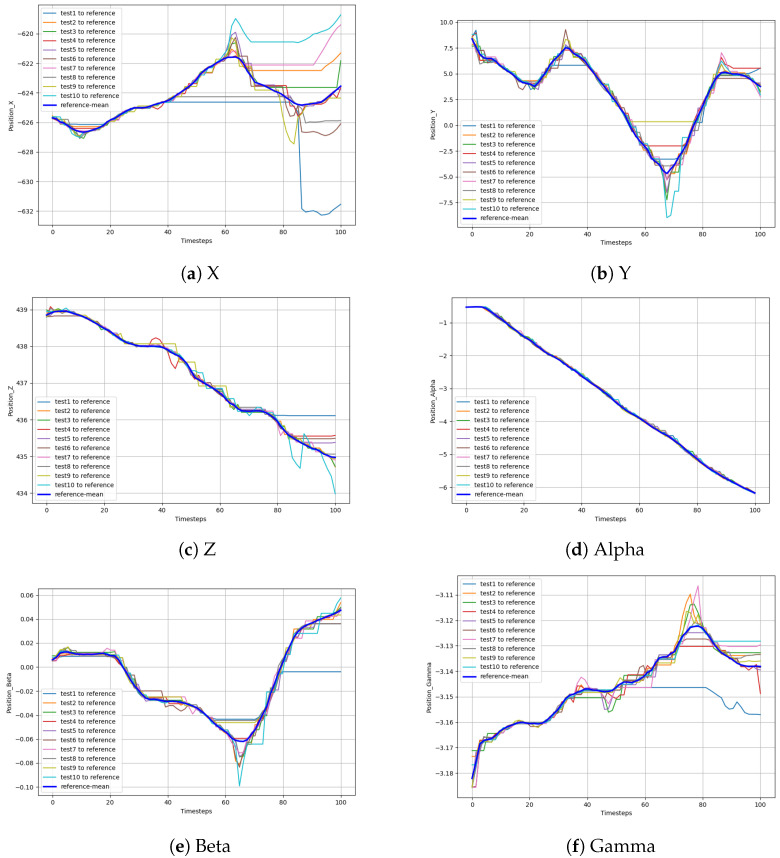
Data for positions X, Y, and Z, and postures Alpha, Beta, Gamma aligned with DTW.

As shown in Figure 7, the pose data of the robot end were collected by dragging the teaching robot ten times and screwing the M30 bolts at 300°. The change trend for each pose datum corresponding to the ten screwing processes was similar. However, due to the different drag teaching speeds, the sequence changed at different speeds. Let us take the mean sequence of the preprocessed data as a reference trajectory. Data alignment was carried out using the DTW method. The aligned path sequence is shown in Figure 8. The 10 curves in each diagram correspond to the robot pose, and the thick blue line represents the average of each corresponding pose. After DTW alignment of the data, all trajectories showed similar trends aligned to the mean curve.

**Figure 9:**
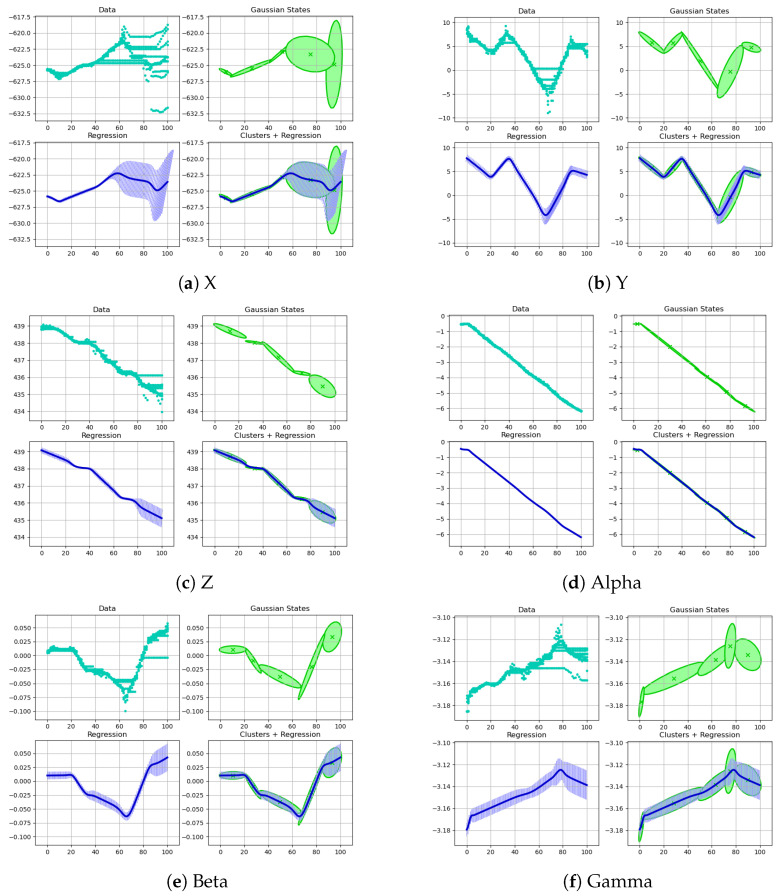
Data positions X, Y, and Z, and postures Alpha, Beta, and Gamma aligned with GMM-GMR.

The DTW-aligned trajectories were clustered and regressed with GMM-GMR. The parameter that GMM needs to determine is $\left\{K,\pi_{k},\mu_{k},\sum_{k}\right\}$. Given the ten types of teaching data collected, K=5 was taken, and then the parameter $\left\{\pi_{k},\mu_{k},\sum_{k}\right\}$ was estimated using the EM algorithm. The DTW-aligned data were clustered with a parameterized GMM model. Then, we used GMR to find the covariance and mean and fitted a trajectory. Figure 9 shows the characteristic encoding trajectory of the screw. The green dot in each subgraph represents the model input, and the green curve represents the encoded end pose. The blue curve encoded by GMR represents the covariance (light blue) and the mean (dark blue), i.e., the mean and variance features of multiple presentations extracted after GMM-GMR processing.

**Figure 10:**
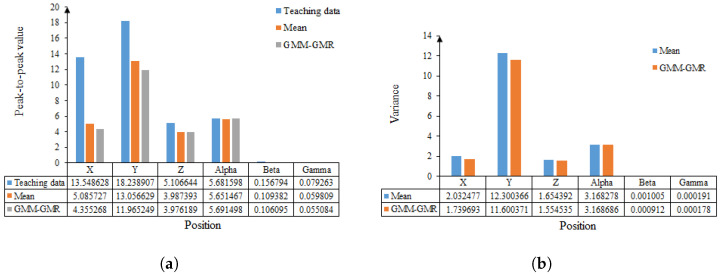
Statistical analysis of data at positions X, Y, and Z, and postures Alpha, Beta, and Gamma. (**a**) Comparison of pose peaks and peaks of the three methods. (**b**) Comparison of data variance obtained from mean and GMM-GMR.

Figure 10a is the peak-to-peak comparison of the pose data obtained by the three methods. Among them, the blue square column represents the dragging and teaching data, and these data fluctuate the most. The orange data represent the average values of the solved pose data, which are smaller than the taught data’s peak-to-peak values. The grey square bars show data from GMM-GMR. According to Figure 9, it can be seen that the data obtained by GMM-GMR and the data obtained by averaging are very close at Alpha. In the other data, the peak-to-peak value of the trajectory calculated by GMM-GMR is smaller and smoother.

In Figure 10b, the blue bar is the variance obtained by taking the mean value of the teaching data, and the orange bar represents the variance of the data. Through comparison, it can be seen that the variance of the data obtained using GMM-GMR was small and the trajectory is smoother. The resulting trajectories were sent to the robot to reproduce the twisting process. According to the trajectory obtained by GMM-GMR, it was found that the robot tightening is smoother. It was verified that the robot could successfully complete the twist by changing the starting point.

Verification of the reproducibility and generalization was accomplished by transforming different bolt twists. M30 × 3.5 nylon bolts were selected to verify the reproduction of the teaching track. Selecting M24 × 3 nylon bolts and steel bolts, and M20 × 2.5 nylon bolts and steel bolts, for the reproduction and generalization experiments, it was proven that the learned model could complete the recurrence and generalization screwing tasks.

**Figure 11:**
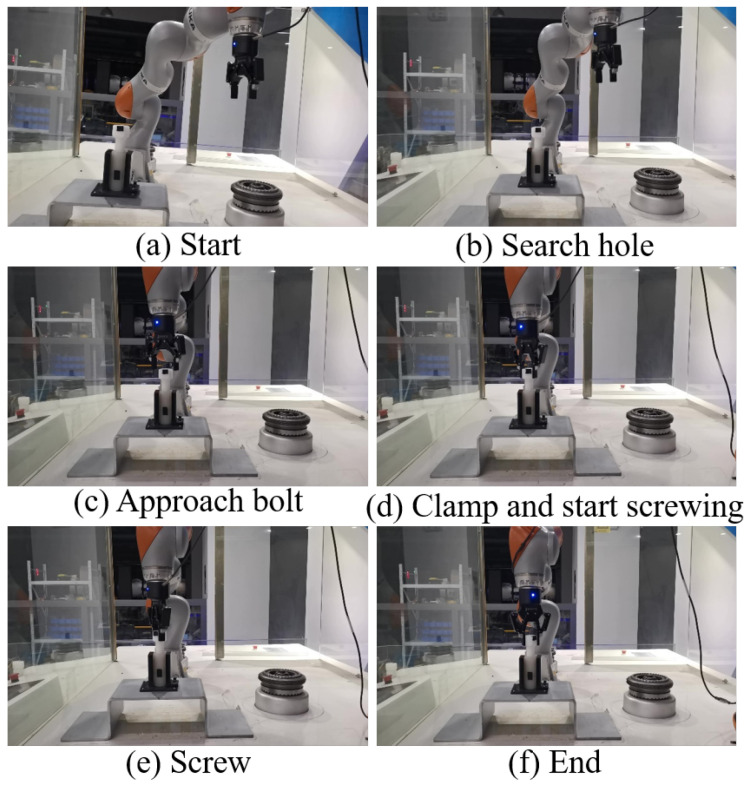
Process diagram of the bolt screwing assembly experiment.

During the experiment on the robot’s real target tendency, it was found that the robot had errors. After many experiments, it was found that the robot could only reach 8 mm less than the specified point on the z-axis coordinate each time during trajectory planning. After the path planned by the DMP, we added an 8 mm adjustment to each z-axis coordinate. There was still a small gap between the actual DMP planning target point and the starting point of the screw, using teaching alignment or other sensor calibration methods. We determined the difference between the two points and moved it. The experimental process is shown in Figure 11. The robot with the two-finger gripper fixed at the end gradually approached the bolt from the starting point and closed the gripper. Then, the robot clamped the bolt and twisted it clockwise. Under the action of the DMP hole-finding trajectory learning and the generalized screwing trajectory of GMM-GMR, the robot could successfully complete the hole-finding and screwing operations.

### 4.2. Generalization Experiment of Plastic Bottle Cap Trend and Screwing

**Figure 12:**
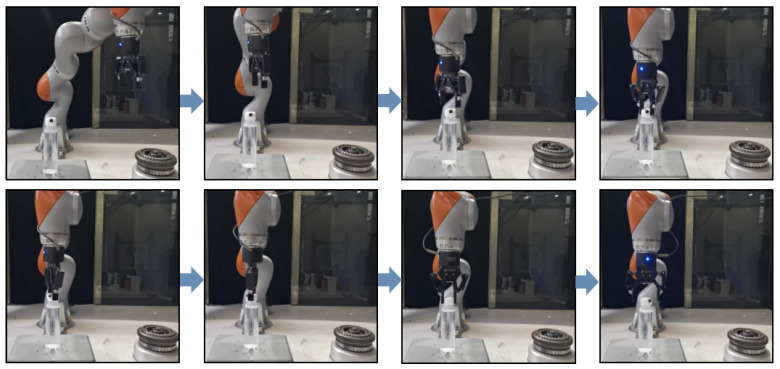
Experimental process diagram of the robot screwing a bottle cap.

A cap was placed on a bottle. The coordinate of the end position of the robot under the base frame was obtained. We recorded the teaching starting point, calculated the transition matrix from the original teaching starting point to the current screwing starting point, and superimposed it onto the original matrix. Generalization of the screwing object was performed. From the target point planning of the screwing target trend trajectory to the starting point of screwing, after reaching this point, the bottle cap was screwed in one circle. The start point and target point of the generalized trajectory were input into the DMP model. The DMP-based teaching trajectory learning and generalization errors were small and we could maintain a similar trend to the original trajectory in the experiments. For the screwing part, a transfer matrix of the two tasks was obtained, and the original teaching screwing starting point became the new screwing starting point. The rotation angle was not changed. The transition matrix is shown in (12):

(14) $$\left\{\begin{array}{c} S_{current}=S_{original}+T\\ T=S_{current0}-S_{original0} \end{array}\right.$$

where *S* represents the pose matrix. From the transition matrix of the starting point, the position change matrix of the process can be obtained and then superimposed onto the original teaching trajectory to complete the generalization. A generalization experimental diagram of the bottle cap trend and screwing is shown in Figure 12. The robot changed the generalized trajectory of the target point in the DMP model, gradually reached the top of the bottle cap from the starting point, and closed the gripper. Then, the robot reproduced the generalized screwing trajectory of GMM-GMR to complete the screwing of the bottle cap. The results confirmed the effectiveness of DMP in the path learning and generalization of robot screwing hole finding, as well as demonstrating the generalization of the GMM-GMR skill learning method to different screwing objects.

### 4.3. Generalization Experiment for Faucet Target Trend Obstacle Avoidance and Screwing

A platform enabling the robot to turn a faucet was built. An obstacle was set up on the path that the robot was required to pass to reach the faucet, in order to observe the obstacle avoidance effect of the robot’s hole-finding strategy. The position at which the robot could grip the faucet was read as the start point of the screw, i.e., the target point of the screw target trajectory. Generalization of the screw object was performed by superimposing the transfer matrix of the original taught screw starting point onto the new screw starting point and superimposing it onto the original matrix. The faucet could be rotated at an angle of 90°, and the robotic arm could rotate the faucet 90° to complete the experiment.

**Figure 13:**
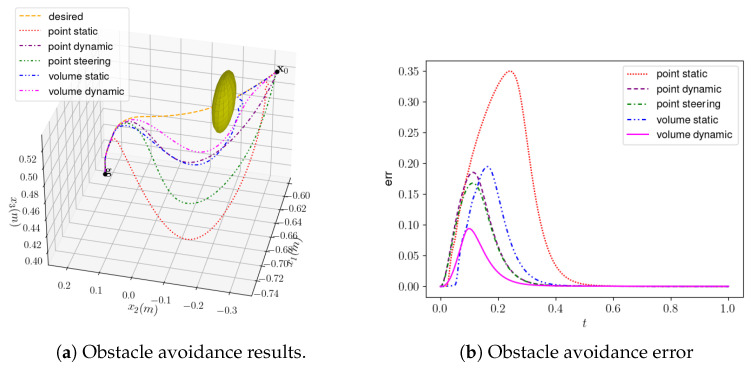
Obstacle avoidance experiment.

Table 2 lists the hyperparameters of each artificial potential field method. The changing target trend point was input into the DMP model, and the end generalization trajectory of the manipulator based on the teaching curve was obtained. The trajectories of target learning and generalization were obtained using DMP. The generalized trajectory had a similar trajectory trend to the original teaching trajectory. The parameters of various artificial potential fields were brought into the hole-finding strategy model based on DMP, and the obtained trend trajectory is shown in Figure 13a. The orange dotted line is the learning curve after transforming the target end point, where A is the starting point, B is the end point reached by the DMP generalization trajectory, and the yellow ellipsoid represents the potential function of the obstacle. The other fuchsia, blue, purple, green, and red dotted lines represent the trajectories planned by the five artificial potential field obstacle avoidance methods, respectively. The smaller the distance of the expected curve of the obstacle avoidance curve planned by different methods, the smaller the error. The errors in planning using different methods are shown in Figure 13a.

**Figure 14:**
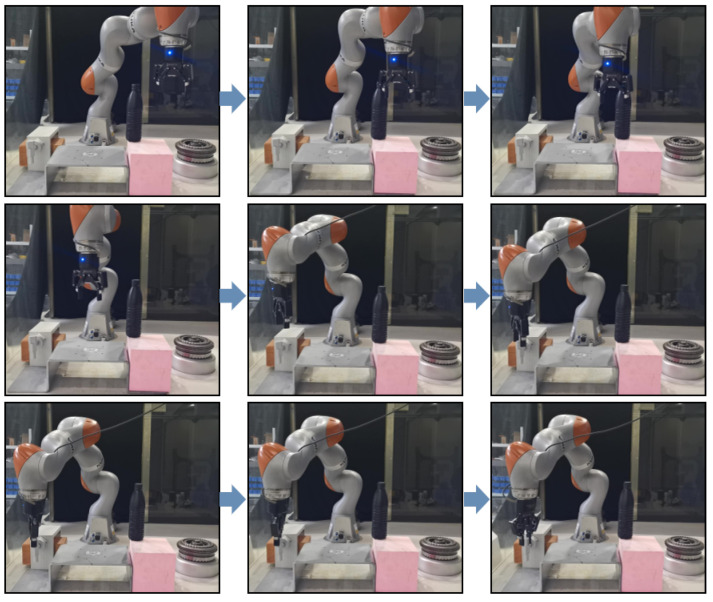
Process diagram of robot turning a faucet.

In the experiment, the limitation methods that do not exceed the robot itself are denoted by the fuchsia curve (dynamic volume obstacle avoidance) and the blue dotted line (static volume obstacle avoidance). The error of dynamic volume obstacle avoidance was the smallest, so the method of dynamic volume obstacle avoidance was used to plan obstacle avoidance and then reach the position above the faucet. The robot reached over the faucet and moved to a position. The transfer matrix of the original teaching screw start point to the current screw start point was calculated and processed and then superimposed onto the original matrix to generalize the screw object. The faucet could be rotated 90° by the robot to complete the rotation experiment. A process diagram is shown in Figure 14. From the starting point to the obstacle range, the robot planed forward and upward movements to avoid the obstacle and tended to move to the top of the faucet to be screwed. The griper was closed, the robot turned 90° clockwise to complete the tap, and then the griper was opened. The robot planed a hole-finding curve with the smallest error for obstacle avoidance through the hole-finding strategy using DMP and the artificial potential field. Then, the generalized screwing curve calculated using GMM-GMR showed that the robot successfully achieved screwing. This experiment verified the effectiveness of the method proposed in this paper and indicated that it is applicable to different screwing objects.

In summary, this section validates the reusability and versatility of the twisting skills obtained through experiments with robots twisting bolts, plastic bottle caps, and faucets, as well as applying twisting experience to different objects to complete operations. The robots could learn the skills demonstrated by humans, generalize to different tasks, and achieve flexible experimental goals for different tasks and task parameters. The experiments showed that the task framework proposed in this paper could achieve the generalization of twisting tasks and improve the efficiency and robustness of robot twisting operations.

## 5. Conclusions

In this study, we focused on improving a robot’s ability to perform screwing tasks in complex environments. Through extensive research and modeling, we made significant advancements in enhancing the robot’s generalization capabilities. We developed a robot screwing platform using the KUKA iiwa 7 R800 robot to validate our findings. Our experiments included tasks such as hole finding in complex environments and learning screwing skills using innovative methods. Through various tasks, including different types of screwing scenarios, we successfully demonstrated the robot’s ability to reuse its screwing experience and generalize it to different objects. Our research contributes to the field of robotics by improving the robot’s adaptability and efficiency in screwing tasks. This has important implications for industry, as it can enhance production efficiency, reduce labor costs, and improve the quality and reliability of screw assembly processes.

In future work, as the rotational learning of position information is insufficient to ensure the flexibility of mechanics, we will focus on the fusion of force information and position information to ensure smoother and safer operations.

## Figures and Tables

**Table 1:** Error results of target trajectory learning and generalization based on DMP.

No.	Target Point	Actual End Point	Error
1	(−682.26, 74.35, 363.78)	(−682.19, 74.71, 363.40)	0.108%
2	(−683.62, −73.51, 361.67)	(−683.51, −69.32, 361.35)	1.148%
3	(−593.05, −72.52, 362.11)	(−595.29, −68.36, 361.78)	1.410%
4	(−595.29, −68.36, 361.78)	(−595.29, 72.66, 359.85)	0.493%

**Table 2:** Setting the hyperparameters of each artificial potential field method.

No.	Method	Hyperparameters
1	Point static	$p_{0}=0.1,\eta =1$
2	Point dynamic	λ=0.2,β=2
3	Point steering	γ=20,β=3
4	Volume static	A=10,η=1
5	Volume dynamic	$\lambda =10,\beta =2,\eta =\frac{1}{2}$

## Data Availability

Data are contained within the article.
